# Practice of the principle of right conduct in obtaining informed consents and legibility of the consent forms - a clinical audit

**DOI:** 10.1186/1753-6561-9-S1-A16

**Published:** 2015-01-14

**Authors:** R Hassan, A Ramli, I Callanan

**Affiliations:** 1 Royal College of Surgeons in Ireland, 123 St. Stephen’s Green, Dublin, Ireland; 2 Clinical Audit Department, St. Vincent's University Hospital, Elm Park, Dublin, Ireland

## Background

Informed consent forms must be clear and include all the necessary information of the possible risks, benefits and complications of the procedure needing consent to. Thus, the form should not include illegible handwriting, medical jargons and abbreviations. Ethically, it should be obtained by a higher ranked physician [1]. In a thirteen-week audit conducted in a Dublin hospital, we observed the effects of revised informed consent forms by quantifying the number of errors, if any, created through this process and propose an immediate solution to it.

## Methods

Retrospective (between 1/11/2012-10/2/2013) and randomised data collection method was applied on 100 informed consent forms found in patient’s files, using a data collection form consisting of 7 questions.

## Results

Out of 100 consent forms, only 32% are new, contain no abbreviations and are written legibly. One form was not filled up, but the procedure associated with it (blood transfusion) was carried out nevertheless. 38% of informed consent was obtained by interns. Similarly, 10 out of 38 consent forms filled up by interns pertain to major procedures in which patients were prescribed a general anaesthesia, which rendered them unconscious. 48% of consent forms contain abbreviations while 77% were legibly written. In 7 cases, the doctors’ names were illegibly written while in 22 cases, the rank of the doctor who obtained the consent could not be identified.

## Conclusions

Our study discovered a low percentage of perfectly filled informed consent forms and high percentage of legible forms. High numbers of consent were obtained by the interns, a practice which did not adhere to the recommended ethical guidelines[1]. We recommend the use of high quality rubber stamps to allow for better readability of the doctors’ name and the introduction of policy that holds the consultants responsible in ensuring the legibility of informed consent forms of their respective patients.
